# Comparison of Feature Selection Methods in Machine Learning Models of Cancer Information Seeking Among United States Adults: Cross-Sectional Study

**DOI:** 10.2196/75862

**Published:** 2026-04-20

**Authors:** Ying Liu, Kesheng Wang

**Affiliations:** 1 Department of Biostatistics and Epidemiology, College of Public Health East Tennessee State University Johnson City, TN United States; 2 Cancer Survivorship Research Center, College of Nursing University of South Carolina Columbia, SC United States; 3 Department of Epidemiology and Biostatistics, Arnold School of Public Health University of South Carolina Columbia, SC United States

**Keywords:** beliefs, cancer information seeking, data mining, feature selection, health behaviors, knowledge, machine learning, PCA, random forest

## Abstract

**Background:**

Feature selection is the process of identifying the most informative and relevant features from a larger set of candidate features in machine learning (ML) models. The Boruta algorithm and the least absolute shrinkage and selection operator (LASSO) are 2 widely used methods.

**Objective:**

This study aimed to (1) compare several feature-selection strategies, including Boruta, LASSO, their intersection, principal component analysis (PCA), and a no–feature-selection baseline, and (2) evaluate ML models to predict cancer information–seeking behavior among US adults.

**Methods:**

Data from 5505 individuals (2630 cancer information seekers and 2875 nonseekers) were selected from the 2022 Health Information National Trends Survey. The Boruta algorithm, LASSO, and PCA were used to perform feature selection of 73 variables. Five ML tools (the support vector machine algorithms, logistic regression [LR], random forest [RF], k-nearest neighbor, and extreme gradient boosting) were applied to develop ML models to predict cancer information–seeking. The area under the receiver operating characteristic curve (AUC) and the DeLong test were used to evaluate and compare the performance of the models. Stepwise LR analysis was performed to estimate the odds ratios and their 95% CIs for the associations of potential variables selected in ML analyses with the outcome.

**Results:**

Overall, 47.8% (2630/5505) of respondents reported seeking cancer information (949/2189, 43.4% of men; 1681/2189, 50.7% of women). RF achieved the highest AUC (0.781) and second-highest accuracy (0.714) using LASSO-selected variables, while the support vector machine with linear kernel and LR models using all 73 features yielded the highest accuracy (0.717). Notably, RF produced comparable AUCs when using Boruta-only features, LASSO-only features, or no feature selection yet (all 73 features); these AUCs were significantly higher than those derived from PCA components or from the 20 PCA-loading–based variables. Stepwise LR confirmed that 19 of the 27 shared variables selected by both Boruta and LASSO were independently associated with information seeking (*P*<.05). The top predictors included a personal history of cancer, greater worry about developing cancer, a family history of cancer, non-Hispanic White race, higher household income, awareness of genetic testing, viewing health-related videos on social media, interest in cancer screening, being offered access to an online medical record, and knowledge of human papillomavirus.

**Conclusions:**

Boruta and LASSO demonstrated strong and consistent performance in feature selection for predicting cancer information seeking, whereas PCA provided a dimension-reduced yet less predictive alternative. Findings offer actionable insights for tailoring public health communication strategies and improving engagement in cancer information resources among US adults.

## Introduction

With the development of science and technology, people seek health information from various sources, including online and offline approaches, for different reasons, such as to understand personal or family members’ health conditions and to make decisions on health professionals’ recommendations [1]. According to the United States National Cancer Institute (NCI) report, approximately 40.5% of people will have cancer in their lifetime based on 2017-2019 data. Over 2 million people were diagnosed in the United States in 2024 [2]. Patients often actively seek cancer information to better understand their diagnosis, treatment options, and home care. Their family members frequently join in the search for additional information. Healthy people may seek cancer information to enlarge their knowledge and improve their quality of life, such as changing their diet to healthy food and having regular physical exams [3]. Generally, cancer information–seeking behavior may increase knowledge, preventive behaviors, and screening behaviors; moreover, cancer information seekers may be more likely to adopt healthy lifestyle behaviors and get screened for cancer [3-7].

Several sociodemographic factors, such as being female, aged 55-64 years vs 40-44 years, having higher education, identifying as Black or Hispanic, and being married, have been positively associated with cancer information seeking in the US population. Racial disparities and variations by marital status and cancer status have also been reported [5,8-13]. Furthermore, behaviors such as alcohol use and tobacco smoking use, as well as certain chronic conditions (eg, cancer and anxiety), may influence cancer information seeking [3,14-16]. Beliefs about cancer and knowledge of genetic testing have also been associated with cancer information–seeking behavior, though findings in these areas remain inconsistent [13,15,17-22].

Machine learning (ML) and predictive analytics are commonly used in many areas, and they can transform data into useful insights for better understanding and faster decision-making. ML methods can address high-dimensional data, model the etiological and clinical heterogeneity, and translate univariate variable findings into clinically useful multivariate decision support systems [23-27]. Feature selection is a critical step in ML, not only to reduce the dimensionality of the feature space, but also to reveal the most relevant features without losing too much information [28-30]. Feature selection, as a preprocessing stage, is essentially the process of picking some informative and relevant features from a larger collection of features that produce a better characterization of patterns of multiple classes [28,31]. Several feature-selection methods have been used in ML, such as the least absolute shrinkage and selection operator (LASSO) [32-35] and the Boruta algorithm [32,33]. Principal component analysis (PCA) is an unsupervised feature reduction technique that explains the variance-covariance structure of a set of variables through linear combinations known as principal components (PCs) or factors. PCA has been used to reduce the dimensions, but it is not a feature-selection method because all variables remain in each factor [31,36-38].

Numerous studies have investigated cancer information–seeking behaviors [3,8-12,14,19,39]. However, relatively limited research has applied ML approaches to systematically identify key factors associated with cancer information–seeking behaviors. The Health Information National Trends Survey (HINTS) conducted by the NCI is a nationally representative cross-sectional survey of civilian noninstitutionalized adults aged 18 years and older in the United States. HINTS collected comprehensive data on the access to, use of, and needs for health- and cancer-related information, as well as knowledge, perceptions, attitudes, and related health behaviors. It has been widely used to address cancer information seeking [3,8-11,13-20]. Several ML tools, such as logistic regression (LR), support vector machine (SVM), random forest (RF), k-nearest neighbor (KNN), and extreme gradient boosting (XGBoost), have been used in the classification of binge drinking, e-cigarette use, and severe psychological distress using HINTS data [40-43]. Therefore, this study aimed to (1) compare feature-selection methods—Boruta, LASSO, combination of Boruta and LASSO, and PCA-based methods—and (2) develop ML tools to predict cancer information seeking among US adults using the data from the 2022 Health Information National Trends Survey (HINTS 6).

## Methods

### Sample

The data for this study were selected from the HINTS 6, which included 6252 respondents. The HINTS is a nationally representative survey administered by the NCI since 2003. The HINTS targets adults aged 18 years or older in the civilian noninstitutionalized population of the United States. The HINTS, sponsored by the NCI, provides a unique opportunity to explore the characteristics of information seekers and nonseekers, as well as the content of information being sought by the public in a nationally representative sample. Data collection for HINTS 6 started on March 7, 2022, and concluded on November 8, 2022. The overall household response rate, based on the next-birthday method, was 28.1%.

### Ethical Considerations

The original HINTS 6 data collection by the NCI was designated “exempt research” under 45 CFR 46.104 and approved by the Westat Institutional Review Board on May 10, 2021 (project #6632.03.51), with a subsequent amendment approved on November 24, 2021 (amendment ID #3597). This study is a secondary analysis of HINTS 6, a publicly available, deidentified dataset. In accordance with US federal regulations (45 CFR 46) and institutional policies, secondary analyses of deidentified, publicly available data do not require additional institutional review board review. Additional details about the informed consent process, incentives, and methodology can be found in the HINTS 6 methodology report [44].

### Outcome Variable

Individuals were classified as cancer information seekers if they responded “yes” to the question “Have you ever looked for information about cancer from any source?” and those who responded “no” were classified as nonseekers.

### Data Processing of Predictors

A total of 87 predictive variables (including demographic factors, alcohol and tobacco use, health care, medical record, chronic diseases, beliefs about cancer, social media, health and nutrition, etc) were included in the initial analysis. Previous simulation and real data analyses revealed that statistical analysis is likely to be biased if the percentage of missing values is more than 10% [45-47]. Therefore, variables with a missing value rate higher than 10% were removed for further analysis. Finally, 74 variables, including the outcome, were left. After excluding individuals with missing data on the outcome, age, gender, or race, the final sample size was 5505.

Demographic characteristics included gender, age group (18-49 years, 50-64 years, 65-74 years, and 75 years or older), race, education, full-time work (yes or no), income, and health insurance (yes or no). Race was recoded as Hispanic, non-Hispanic White, non-Hispanic Black or African American, non-Hispanic Asian, and other. Education had 4 categories (less than high school, some college, bachelor’s degree, and postbaccalaureate degree). The 4 categories of annual income were <US $19,999, US $20,000-US $49,999, US $50,000-US $74,999, and ≥US $75,000. Table 1 lists the demographic variables.

**Table 1:** Prevalence of cancer information seeking across demographic factors.

Variable		Total, n	Seeking, n	Prevalence, %^a^	*P* value^b^
**Gender**					
	Male	2189	949	43.4	<.001
	Female	3316	1681	50.7	
**Age group**					
	18-49 years	1935	895	46.3	.01
	50-64 years	1607	791	49.2	
	65-74 years	1234	623	50.5	
	>75 years	729	321	44.0	
**Race**					
	Non-Hispanic White	3175	1738	54.7	<.001
	Non-Hispanic African American	878	314	35.8	
	Hispanic	984	371	37.7	
	Non-Hispanic Asian	286	120	42.0	
	Other	182	87	47.8	
**Education^c^**					
	Less than high school	1300	375	28.8	<.001
	Some college	1574	721	45.8	
	Bachelor’s degree	1550	843	54.4	
	Postbaccalaureate degree	1070	687	64.2	
**Income (US $)**					
	<19,999	904	271	30.0	<.001
	20,000-49,999	1431	569	39.8	
	50,000-74,999	946	488	51.6	
	>75,000	2224	1302	58.5	
	Overall	5505	2630	47.8	

^a^The prevalence is the ratio of seeking and total.

^b^*P* value is based on the chi-square test.

^c^11 participants had missing values in the education variable.

**Figure 1:**
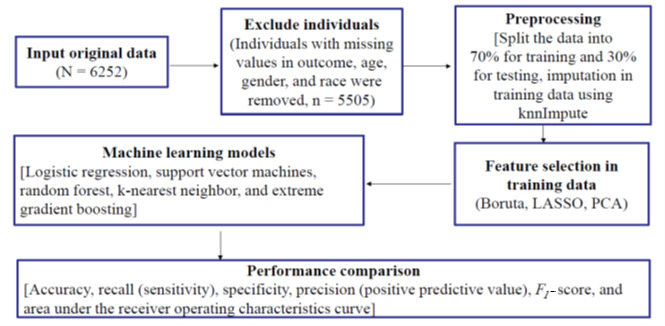
Overview of data curation and machine learning workflow. LASSO: least absolute shrinkage and selection operator; PCA: principal component analysis.

Race was generated for dummy variables. Other predictive variables were binary, ordinal, or continuous. In this dataset, 70% (3854/5505) of the entries were used for training the models, leaving the remaining 30% (1651/5505) for the testing set. The missing values were imputed using the “knnImpute” method in caret based on the training data only [48]. The derived imputation values were subsequently applied to the test data. The full list of 73 predicting variables is listed in Multimedia Appendix 1. Figure 1 shows an overview of the data curation and ML process.

### Feature-Selection Methods

Feature selection was performed within the training data. The Boruta algorithm, implemented in R software (version 4.5.2; R Foundation for Statistical Computing) via the “Boruta” package, automatically performs feature selection on a dataset using an RF classifier [49]. The LASSO feature selection was applied using the “glmnet” package in R software [50]. This method regularizes the model by applying a penalty (λ), shrinking the regression coefficients, and reducing some of them to 0. The feature-selection phase occurs after the shrinkage, where non-0 values are selected as model parameters.

The PCA is a dimensionality reduction technique that transforms a set of correlated variables into a smaller set of uncorrelated variables called PCs. Generally, the first PC will be the linear combination of the variables that captures the maximum amount of information in the data and will be correlated with at least some of the observed variables, while the second PC identified accounts for the second-largest amount of variance in the data and is uncorrelated with the first PC, and so on. Eigenvalues indicate the amount of variance explained by each PC. A scree plot was used to visualize eigenvalues. The eigenvalue-one criterion (eigenvalue ≥1) is commonly used to decide how many PCs to retain. Eigenvectors are the weights used to calculate PC scores. The PC score is a linear combination of observed variables weighted by eigenvectors. Assume there are n individuals and k observed predictors included in the PCA, then there are k PCs in total. The equation of the PC score for each individual can be written as:

PC_ij_ = b_1j_x_i1_ + b_2j_x_i2_ + ... + b_qj_x_iq_

where

PC_ij_ = the jth PC score for the ith individual, i = 1, 2, ..., n; j = 1, 2, ..., k

b_kj_ = the regression coefficient for observed variable k in _the_ jth PC

X_ik_ = the value of individual i on the observed variable k

q = the number of PCs, q = 1, 2, ..., k

In PCA, the factor loading of a variable represents the correlation between the original variable and a given PC. The loading indicates how much each original variable contributes to a specific PC. Large absolute values of loadings indicate that the corresponding variable has a strong relationship with that particular PC. A factor loading of a variable is considered large if its absolute value exceeds 50%. The PCA was performed using SPSS software (version 31; IBM Corp). For ML analysis, the PC scores for each individual were initially used as predictors. Furthermore, from each of the uncorrelated PCs, 1 variable with the highest loading/correlation coefficient with the PC was chosen for further ML analysis.

### ML Methods

A total of 5 ML algorithms were used, including LR, SVM, RF, KNN, and XGBoost. The caret package, incorporating other packages in R, was used for LR, KNN, RF, SVM, and XGBoost [48]. A 10-fold cross-validation approach was applied, and multiple parameters for each algorithm were optimized using a grid search.

For the LR model, the “glmnet” in the caret package was used. In the grid search, we set alpha = 0:1 and lambda = seq(0.001, 1, length = 10).

The SVM algorithm includes linear kernel and radial kernel [51]. In the grid search, we set C = c(0.01, 0.1, 0.2, 0.5, 1, 2)) for the linear kernel; sigma = c(0.05, 0.25, 0.5, 1, 2) and C = c(0.05, 0.25, 0.5, 1, 2)) for the radial kernel.

The RF algorithm randomly selects a subset of variables to construct multiple decision trees (DTs) [52,53]. In the grid search, we set mtry = c(1:15) and ntree = 300, where the mtry parameter refers to the number of variables used in each random tree, while ntree refers to the number of trees that the forest contains. The mtry range (1-15) was chosen to cover and exceed the conventional default (√p ≈ 8-9) while limiting overfitting and computational burden.

KNN, a simple ML algorithm based on a clustering algorithm with supervised learning, calculates the average of the numerical target of the k-nearest neighbors [54]. KNN is more suitable for low-dimensional data with a small number of input variables. In the grid search, we set k=1:20.

XGBoost [55] is a supervised ML method for regression and classification tasks similar to the RF classifier. In the grid search, we set the nrounds = c(200,300), max_depth = c(6, 10, 20), colsample_bytree = c(0.5, 1.0), eta = c(0.1, 0.3), gamma= c(0, 0.5), min_child_weight = c(1,2), and subsample = c(0.75, 1.0).

### Performance of ML

To evaluate the performance of feature-selection methods, we used several metrics, including accuracy, recall (sensitivity), specificity, precision (positive predictive value), *F*_1_-score, and area under the receiver operating characteristic curve (AUC). The R packages used included “caret,” “kernlab,” and “ROCR.”

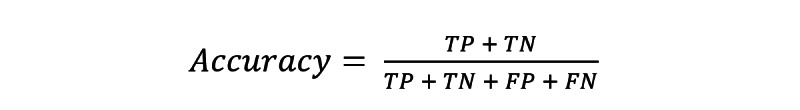

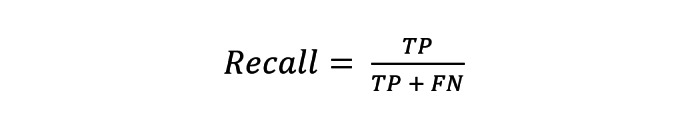

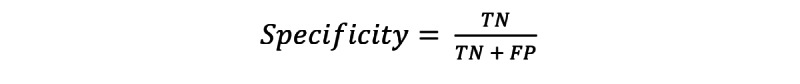

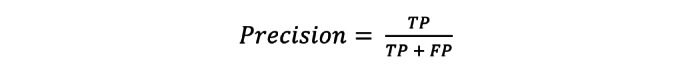

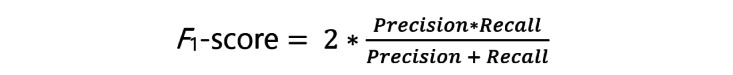











where *TP* is the number of true positives, *TN* is the number of true negatives, *FP* is the number of false positives, and *FN* is the number of false negatives. The *F*_1_-score is a harmonic mean that combines both recall and precision. The DeLong test was used to compare the statistical differences of the AUC between different models [56].

### Statistical Analysis

The categorical variables were presented in their raw values along with the proportions for categorical variables. The chi-square test was used to examine the associations of categorical variables with cancer information seeking across demographic variables. Stepwise LR analysis was performed to estimate the odds ratios (ORs) and their 95% CIs for the associations of potential factors selected in ML analyses with the outcome. All statistical analyses were performed using SPSS software (version 31).

## Results

### Prevalence of Cancer Information Seeking

Among the 5505 adult respondents, 2630 were classified as cancer information seekers and 2875 as nonseekers (Table 1). The overall prevalence was 47.8% (2630/5505; 949/2189, 43.4% for men and 1681/2189, 50.7% for women). The prevalence increased with age (895/1935, 46.3%; 791/1607, 49.2%; 623/1234, 50.5% for age groups 18-49, 50-64, and 65-74 years, respectively). The age group of >75 years had a lower prevalence (321/729, 44%). The prevalence was higher in those with higher education and higher income.

### Feature Selection

**Figure 2:**
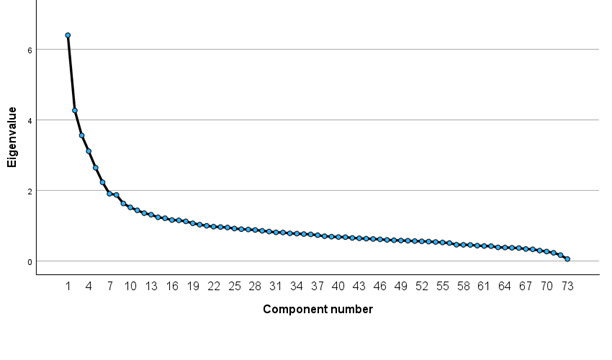
Scree plot showing 20 principal components with eigenvalues higher than 1.

The Boruta algorithm selected 43 variables, and LASSO selected 42 variables related to cancer information seeking (Multimedia Appendix 1). Of these, 27 variables were identified by both methods. The PCA identified 20 uncorrelated PCs with eigenvalues >1 (Figure 2 and Multimedia Appendix 2). From each PC, we selected the variable with the highest loading/correlation (Multimedia Appendix 3).

### ML Performance

**Figure 3:**
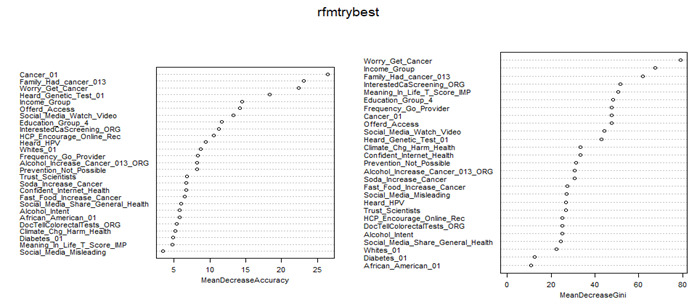
Plot of mean decrease accuracy (left panel) and mean decrease Gini (right panel) values using the random forest algorithm and 27 variables selected by both Boruta and least absolute shrinkage and selection operator.

**Figure 4:**
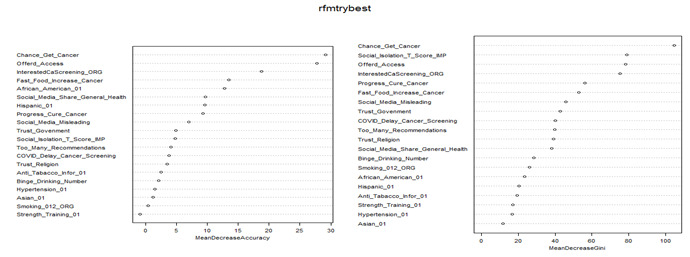
Plot of mean decrease accuracy (left panel) and mean decrease Gini (right panel) values using the random forest algorithm and 20 variables with the highest loading from 20 PCs.

The performance statistics are summarized in Multimedia Appendix 4. RF achieved the highest AUC (0.781) and second-highest accuracy (0.714) using 42 LASSO-selected variables, while using all 73 features yielded the highest accuracy (0.717). Using the 27 common variables identified by both Boruta and LASSO, the RF model achieved the highest predictive accuracy (0.711), the same as using SVM with a linear kernel, closely followed by SVM with a radial basis function (RBF) kernel (0.708) and LR (0.708). When using 20 PC scores and selecting 1 variable from each of 20 PCs with the highest loading with the PC, the RF models showed lower accuracy (0.694 and 0.678, respectively). In Figure 3, the mean decrease in accuracy and the mean decrease in Gini metrics from the RF algorithm are shown for the 27 variables identified by LASSO and Boruta. Figure 4 displays the same metrics for the 20 variables derived from each PC. Based on the Gini values in Figure 3, the strongest predictors included cancer-related worry, income, a family history of cancer, interest in cancer screening, meaning and purpose in life, education, more frequent provider visits, having a cancer diagnosis, being offered access to an online medical record, watching health-related videos on social media, and awareness of genetic testing. Plot of mean decrease accuracy and mean decrease Gini values using RF algorithm, XGBoost, gradient boosting machine, LR models, and the 21 factors are illustrated in Figures S1-S4 in Multimedia Appendix 5.

**Figure 5:**
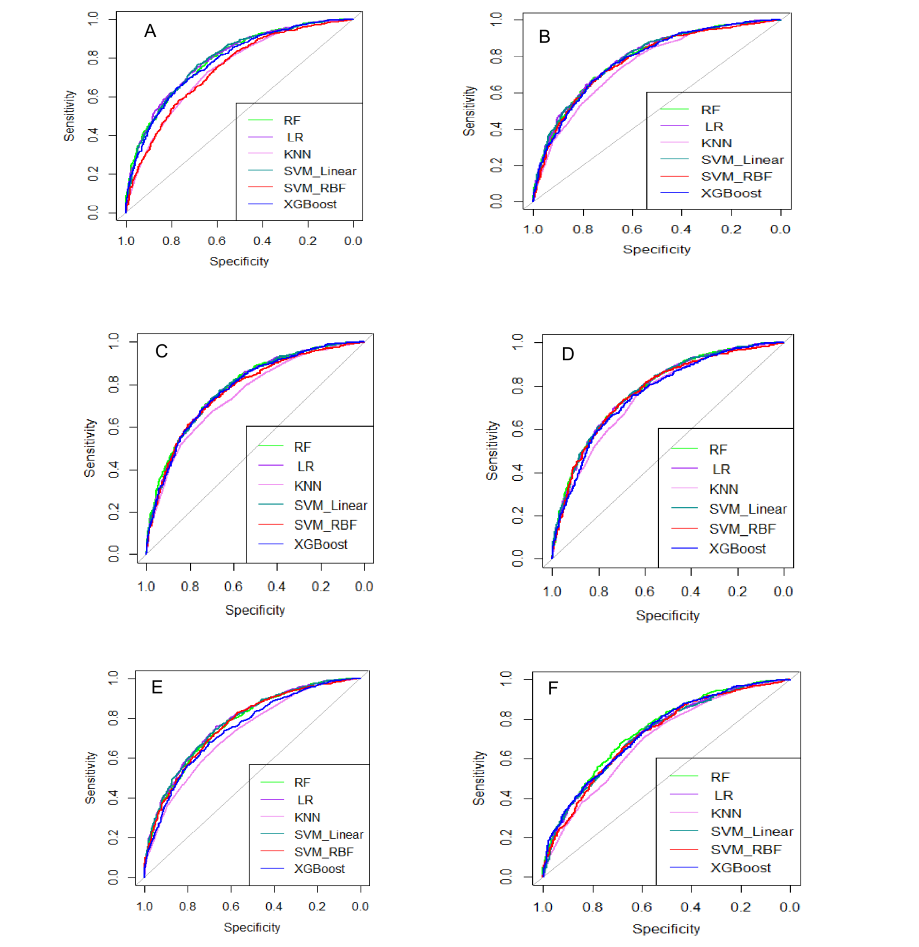
AUC curves in the test data in comparison of RF with other five ML models. (a) All features, (b) LASSO, (c) Boruta, (d) LASSO and Boruta, (e) 20 PCA scores, (f) 20 highest loading variables from 20 PCs.

To determine whether the RF model had a significantly higher AUC than other models, the DeLong test was used. The AUC differences between RF and other models, 95% CIs of the AUC difference, and *P* values are illustrated in Table 2. The RF model yielded comparable AUC values with SVM with a linear kernel and LR, except for using PCA-based 20 variables (*P*>.05). In contrast, the AUCs from these RF models were significantly higher than those obtained using SVM with an RBF kernel, KNN, and XGBoost (*P*<.05), except for SVM with an RBF kernel using 20 PC scores and XGBoost using LASSO and PCA-based 20 variables. Figure 5 presents the AUC in the test data for each ML model across the 6 feature-selection methods.

**Table 2:** Comparison of machine learning models with random forest using the DeLong test of area under the receiver operating characteristic curve (AUC) difference.

Features	SVM^a^_Linear		SVM_RBF^b^			LR^c^			KNN^d^			XGBoost^e^	
	AUC difference (95% CI)	*P* value	AUC difference (95% CI)	*P* value	AUC difference (95% CI)		*P* value	AUC difference (95% CI)		*P* value	AUC difference (95% CI)		*P* value
All features	0.0007 (–0.0101 to 0.0115)	.89	0.0509 (0.0359 to 0.0659)	<.001^f^	–0.0012 (–0.0098 to 0.0074)		.78	0.0464 (0.0306 to 0.0622)		<.001^f^	0.0109 (0.0015 to 0.0204)		.02^f^
LASSO^g^	–0.0019 (–0.0113 to 0.0075)	.69	0.0117 (0.0014 to 0.0219)	.03^f^	–0.0044 (–0.0135 to 0.0047)		.34	0.0288 (0.0156 to 0.0420)		<.001^f^	0.0057 (–0.0037 to 0.0152)		.24
Boruta	0.0057 (–0.0037 to 0.0151)	.23	0.0165 (0.0054 to 0.0276)	.004^f^	0.0048 (–0.0045 to 0.0142)		.31	0.0373 (0.0228 to 0.0519)		<.001^f^	0.0090 (0.0002 to 0.0179)		.04^f^
LASSO and Boruta	0.0005 (–0.0074 to 0.0084)	.91	0.0091 (0.0007 to 0.0174)	.04^f^	0.0005 (–0.0078 to 0.0088)		.90	0.0256 (0.0132 to 0.0380)		<.001^f^	0.0182 (0.0089 to 0.0207)		.001^f^
PCA^h^ score	–0.0082 (–0.0190 to 0.0026)	.14	0.0032 (–0.0078 to 0.0143)	.57	–0.0089 (–0.0197 to 0.0019)		.11	0.0438 (0.0278 to 0.0599)		<.001^f^	0.0278 (0.0117 to 0.0338)		<.001^f^
PCA (highest loading variable)	0.0189 (0.0084 to 0.0293)	<.001^f^	0.0238 (0.0133 to 0.0342)	<.001^f^	0.0173 (0.0070 to 0.0276)		.001^f^	0.0469 (0.0303 to 0.0635)		<.001^f^	0.0105 (–0.0029 to 0.0238)		.13

^a^SVM: Support vector machine.

^b^RBF: Radial basis function.

^c^LR: Logistic regression.

^d^KNN: k-nearest neighbor.

^e^XGBoost: Extreme gradient boosting.

^f^*P*<.05 based on DeLong test.

^g^LASSO: The least absolute shrinkage and selection operator.

^h^PCA: Principal component analysis.

**Figure 6:**
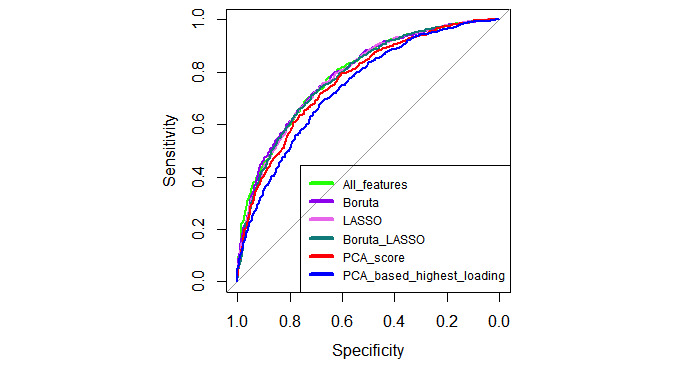
Area under the receiver operating characteristic curve in the test data using the random forest model for feature-selection methods. LASSO: least absolute shrinkage and selection operator; PCA: principal component analysis.

To evaluate whether differences exist among feature-selection methods, the RF model and the DeLong test were used. The pairwise AUC differences among 6 methods, 95% CIs of the AUC difference, and *P* values are illustrated in Table 3. Boruta, LASSO, and using all 73 features did not show significant differences (*P*>.05), whereas these methods showed higher AUC than methods using PCA score and PCA-based selection of highest loading variables (*P*<.05). The combined method of Boruta and LASSO did not show a significant difference from LASSO-only, whereas it showed lower AUC than methods using all 73 features and Boruta, but higher AUC than methods using PCA score and PCA-based selection of highest loading variables (*P*<.05). Furthermore, using PCA scores had a higher AUC than using 20 PCA-based variables (*P*<.05). AUC in the test data for all ML models across the 6 feature-selection methods are illustrated in Figure 6.

**Table 3:** Comparison of feature-selection methods using random forest and the DeLong test of area under the receiver operating characteristic curve (AUC) difference.

Features	Boruta		LASSO^a^		Boruta and LASSO		PCA^b^ score		PCA (highest loading variable)	
	AUC difference (95% CI)	*P* value	AUC difference (95% CI)	*P* value	AUC difference (95% CI)	*P* value	AUC difference (95% CI)	*P* value	AUC difference (95% CI)	*P* value
All features	0.0011 (–0.0035 to 0.0057)	.64	0.0054 (–0.00086 to 0.0116)	.09	0.0080 (0.0013 to 0.0146)	.02^c^	0.0230 (0.0109 to 0.0351)	<.001^c^	0.0492 (0.0339 to 0.0644)	<.001^c^
Boruta	—^d^	—	0.0043 (–0.0028 to 0.0114)	.23	0.0069 (0.0012 to 0.0125)	.02^c^	0.0219 (0.0093 to 0.0345)	<.001^c^	0.0481 (0.0315 to 0.0646)	<.001^c^
LASSO	—	—	—	—	0.0026 (–0.0028 to 0.0079)	.35	0.0176 (0.0050 to 0.0303)	.006^c^	0.0438 (0.0274 to 0.0601)	<.001^c^
LASSO and Boruta	—	—	—	—	—	—	0.0150 (0.0018 to 0.0283)	.03^c^	0.0412 (0.0239 to 0.0583)	<.001^c^
PCA score	—	—	—	—	—	—	—	—	0.0262 (0.0081 to 0.0442)	.005^c^

^a^LASSO: least absolute shrinkage and selection operator.

^b^PCA: principal component analysis.

^c^*P*<.05 based on DeLong test.

^d^Not applicable.

### Stepwise LR Analysis

For the stepwise LR, we used the single training data, where both the Boruta algorithm and LASSO selected 27 common variables for cancer information seeking. Stepwise LR of the 27 variables identified by both Boruta and LASSO, and further confirmed that 19 variables were associated with cancer information seeking (Table 4). Based on ORs, the top predictors included having a cancer diagnosis (OR 1.46, 95% CI 1.35-1.59), greater worry about developing cancer (OR 1.41, 95% CI 1.31-1.53), a family history of cancer (OR 1.35, 95% CI 1.25-1.47), White (OR 1.25, 95% CI 1.12-1.32), having a higher household income (OR 1.24, 95% CI 1.13-1.35), having heard of genetic testing (OR 1.24, 95% CI 1.14-1.35), watching health-related videos on social media (OR 1.24, 95% CI 1.15-1.34), interest in cancer screening (OR 1.20, 95% CI 1.11-1.29), being offered access to an online medical record (OR 1.17, 95% CI 1.08-1.27), and knowledge of human papillomavirus (HPV; OR 1.17, 95% CI 1.08-1.27).

**Table 4:** Stepwise logistic regression analyses of 27 variables.

Variable	aOR^a^ (95% CI)	*P* value
Race (White; reference=other races)	1.25 (1.12-1.32)	<.001
Education (high school or above; reference=less than high school)	1.16 (1.06-1.26)	.001
Income (1-4; 4=>US $75,000)	1.24 (1.13-1.35)	<.001
Alcohol_increase_cancer (1-4; 4=a lot)	1.16 (1.08-1.26)	<.001
Confident_internet_health resource (1-5; 5=very confident)	1.12 (1.03-1.21)	.007
Diabetes (yes; reference=no)	0.91 (0.84-0.99)	.02
Cancer (yes; reference=no)	1.46 (1.35-1.59)	<.001
Family had cancer (yes; reference=no)	1.35 (1.25-1.47)	<.001
Heard_genetic_test (yes; reference=no)	1.24 (1.14-1.35)	<.001
Heard_HPV (yes; reference=no)	1.17 (1.08-1.27)	<.001
Doctor told colorectal cancer tests (yes; reference=never)	1.12 (1.04-1.21)	.004
Interested in cancer screening (0-4; 4=very)	1.20 (1.11-1.29)	<.001
Prevention of cancer not possible (1-4; 4=strongly agree)	1.16 (1.07-1.25)	<.001
Worry_get_cancer (1-5; 5=extremely)	1.41 (1.31-1.53)	<.001
Frequency_go_provider last year (1-6; 6=10 or more times)	1.12 (1.04-1.21)	.004
Offer_access online medical record (yes; reference=no)	1.17 (1.08-1.27)	<.001
Meaning in life (1-5; 5=a lot)	1.09 (1.01-1.18)	.03
Social_media_misleading health information (1-5; 5=a lot)	1.09 (1.01-1.17)	.03
Social_media_watch_video (1-5; 5=almost everyday)	1.24 (1.15-1.34)	<.001

^a^aOR: adjusted odds ratio

## Discussion

### Principal Findings

This study compared 3 feature-selection approaches, including Boruta, LASSO, and PCA, and their variations across 5 ML models to predict cancer information–seeking behavior. Both Boruta and LASSO identified the same set of 27 variables, whereas PCA produced 20 uncorrelated PCs. Based on AUC, the RF model emerged as the best-performing algorithm, yielding comparable AUC values when using Boruta-selected features, LASSO-selected features, or no feature selection at all. In addition, a stepwise LR using the 27 variables identified by both Boruta and LASSO confirmed that 19 variables were significantly associated with cancer information seeking (*P*<.05). The top predictors included having a cancer diagnosis, prior awareness of genetic testing, higher household income, a family history of cancer, being offered access to an online medical record, knowledge of HPV, worry about developing cancer, watching health-related videos on social media, and interest in cancer screening.

### Comparison to Prior Work

Feature selection is a critical step in ML to reduce the dimensionality while retaining the most relevant features without excessive information loss [28-30]. Previous studies have used several feature-selection methods, including LASSO, Boruta, and RF [32-35]. In this study, we applied Boruta and LASSO methods and selected 27 overlapping variables (Multimedia Appendix 1 and Figure 3). The Boruta algorithm is a feature-selection method based on RF and considers important variables, but does not account for collinearity while selecting variables, whereas LASSO regression is useful when multicollinearity exists in the model [49,57,58]. The LASSO can be used as a variable selection method because numerous *β* coefficients that are not strongly associated with the outcome are decreased to 0, which is equivalent to removing those variables from the model. However, the disadvantage of LASSO is that it assumes a more restrictive set of assumptions than RF. For example, the LASSO is a linear model, so anything that matters for your outcome that is not linear in the parameters under estimation is at risk of getting eliminated. Most variables selected by LASSO were also selected by Boruta, which independently confirmed the association between those variables and cancer information–seeking behavior. Previous studies have revealed that combining these methods can lead to more robust feature selection, potentially improving model performance and interpretability [59,60]. In addition, PCA has been used to reduce the dimensions, but it is not a feature-selection method because all variables remain in each PC [31,36-38]; however, PCA can help identify the most important features by examining their contributions (loadings) to the PCs. This study initially conducted data mining using PCA and selected important uncorrelated PCs with eigenvalues >1.0, and for ML analysis, the PC scores were used as predictors, whereas all variables remained in each PC. Notably, based on each of the uncorrelated PCs, we performed feature selection by choosing 1 variable with the highest loading/correlation coefficient with the PC for ML analysis. In this study, PCA identified 20 uncorrelated PCs, while 20 features were selected from these PCs for the development of ML models. Boruta, LASSO, and the use of all 73 features did not show significant differences. However, these methods showed higher AUC than methods using PCA scores and PCA-based selection of the highest loading variables. The combined method of Boruta-LASSO did not yield a statistically significant improvement in model performance compared with LASSO alone. Moreover, the Boruta-LASSO method demonstrated lower discrimination than models using all 73 features or Boruta alone, but achieved higher AUC than models based on PCA-derived features or PCA-based selection of variables with the highest loadings. In addition, models using PCA scores directly showed better performance than models constructed using a reduced set of 20 variables selected based on PCA loadings.

ML methods can accommodate a large number of predictors and capture complex relationships among variables, making them well-suited for predicting health information–seeking behaviors. For example, 3 ML algorithms such as LR, SVM, and RF were applied in predicting the information-seeking behavior of clinicians using an electronic medical record system [61]. Furthermore, LASSO was used to predict health information–seeking behaviors [62], and elastic net and LASSO models were used in predicting internet health seeking [63]. Another study used 4 algorithms (ie, RF, SVM, Bayes generalized linear model, gradient boosting, and an ensemble of the individual methods) to identify search terms and patterns that correlate with changes in obesity and overweight prevalence across Africa [64]. However, limited research has applied ML approaches to systematically identify key factors associated with cancer information–seeking behaviors. This study compared 5 ML tools using 10-fold cross-validation and tested multiple parameters for each algorithm using a grid search for optimal performance. Using 42 LASSO-selected variables, RF achieved the highest AUC (0.781) and second-highest accuracy (0.714). Using the 27 common variables identified by both Boruta and LASSO, the RF model achieved the highest predictive accuracy (0.711), the same as using SVM with linear kernel, closely followed by SVM with RBF kernel (0.708) and LR (0.708). Previous studies have shown that RF is one of the best ML tools. For example, 1 previous study evaluated 10 ML classifiers, including LR, linear discriminant analysis, naive Bayes, KNN, SVM with RBF kernel, DT, RF, XGBoost, AdaBoost, and artificial neural network (ANN), and found that the RF model and the SVM model showed the best performance [65]. Another study developed 7 ML models (LR, KNN, SVM, DT, RF, XGBoost, and ANN) and found that RF and ANN models with the same AUC outperformed other models [66]. Another study compared LR, KNN, gradient boosting, XGBoost, RF, multilayer perceptron, and SVM for diagnosing breast cancer and found that RF achieved the maximum accuracy of 90.68% [67]. However, 1 recent study compared 6 ML algorithms (XGBoost, LR, SVM, RF, KNN, and DT) in developing prognostic models for patients with alpha-fetoprotein–positive hepatocellular carcinoma, and the XGBoost model performed the best, and RF was the second best [68]. Another recent study compared 5 ML models (SVM, XGBoost, Gaussian naïve Bayes, adaptive boosting, and RF) and found that XGBoost and RF achieved superior predictive performance, as evidenced by higher AUCs [69], whereas another study did not find differences among LR, SVM, and RF [70]. As can be seen, there are studies in the literature on the use of ML algorithms in the diagnosis of different types of cancers, as well as other diseases and conditions. The comparisons of ML tools may show heterogeneity.

Furthermore, although LASSO was our primary feature-selection method due to its advantages in handling multicollinearity, high-dimensional data, and overfitting, we used stepwise regression as a secondary, confirmatory analysis for several reasons. First, LASSO selects variables by shrinking coefficients through penalization, but it does not provide traditional inferential statistics such as SEs, *P* values, or likelihood-based model comparison, which are still expected in many epidemiologic and clinical research settings. Stepwise regression allowed us to evaluate whether LASSO-identified predictors remained significant under a conventional regression framework. Second, stepwise selection served as a sensitivity analysis, enabling assessment of the robustness and stability of the LASSO-identified variable set. Recent methodological papers recommend combining penalized regression with stepwise or likelihood-ratio–based checks when the goal includes both prediction and interpretation [71,72]. Accordingly, stepwise regression was used not as the primary modeling strategy, but as a supplementary sensitivity analysis to evaluate the robustness and interpretability of the variables selected by LASSO.

Patient with cancer often seek information regarding their diagnosis, treatment options, treatment costs, potential side effects, and the implications for daily life and survival. While physicians remain a primary source of such information, patients frequently turn to additional resources such as the internet and books to supplement their understanding [12]. A previous study has identified that various factors influence cancer information–seeking behaviors, including gender, education levels, income, and cancer type [3]. In this study, ML techniques were used to identify key predictors of cancer information–seeking behavior. Furthermore, 18 out of 27 selected variables from LASSO and Boruta were confirmed to be significantly associated with cancer information seeking by a stepwise LR model (Table 4). Consistent with prior research, variables including higher educational attainment, race/ethnicity, personal cancer history, family history of cancer, cancer-related beliefs, knowledge of genetic testing, and awareness of HPV were included in the analysis [3,5,8-15,18-20,22]. Furthermore, this study expanded the existing literature by identifying several additional predictors of cancer information–seeking behavior. These included beliefs that certain nutritional factors (such as red meat and alcohol consumption) and climate change increase cancer risk, engagement in social media activities, having access to online medical records, more frequent provider visits, and interest in cancer screening.

Social media has become a main platform and important resource for people to obtain and exchange health-related information and advice [73,74], and has become a new channel for promoting cancer prevention [75]. For example, 1 study found that young adults with cancer used social media to connect with cancer peers for support [76]. Another study showed that social media could enable the seeking and sharing of breast cancer–related information, and enhance patient education, communication, engagement, and empowerment [77]. Social media platforms may increase access to health information and decision aids [78]. However, while social media can make health information more accessible, the use of social media for health information seeking can also create the risk of harm through exposure to misinformation. Because misinformation perceptions can affect attitudes and behaviors, a better understanding of the public’s perceptions of health misinformation on social media and their ability to detect it, as well as possible subgroup differences in such perceptions, is needed [79]. However, misinformation and disinformation on social media have become widespread, which can lead to a lack of trust in health information sources and, in turn, lead to negative health outcomes [80]. In this study, 3 variables related to social media use were selected by both Boruta and LASSO (Multimedia Appendix 1), including social_media_share_general_health (1-5, 5 = almost everyday), social_media_watch_video (1-5, 5 = almost everyday), and social_media_misleading (1-5, 5= a lot). Stepwise LR further confirmed these variables, including social_media_watch_video and social_media_misleading (Table 4). These findings highlight that people involved in social media activities have increased odds of seeking cancer information. Furthermore, it has been shown that most social media users perceive some (46%) or a lot (36%) of false or misleading health information on social media using HINTS 6 data [80]. This study added that most social media users with a high prevalence of false and misleading health information on social media are positively seeking cancer information.

This study further added that beliefs about alcohol use causing cancer were associated with increased odds of cancer information seeking. Alcohol consumption increases the risk of several types of cancer, including liver, esophageal, colorectal, and breast cancer; however, public awareness of the association between alcohol use and cancer remains low and varies by type of alcoholic beverage [81-83]. For example, using the HINTS (2020) data, 1 study found that awareness of the alcohol-cancer link was highest for liquor (31.2%), followed by beer (24.9%) and wine (20.3%). More US adults believed wine (10.3%) decreased cancer risk, compared with beer (2.2%) and liquor (1.7%). Most US adults (>50%) reported not knowing how these beverages affected cancer risk [82]. Another study using the HINTS (2020) data found that 34% of those reporting current alcohol consumption believed that drinking wine decreases or has no effect on cancer risk, compared with 20.8% of those reporting no alcohol consumption [84]. A recent study using several HINTS cycle datasets did not find significant differences in diet-related cancer risk awareness and behaviors between cancer survivors and those without a history of cancer [85]. Among the European Union general population, awareness of the link between alcohol and breast cancer ranged between 10% and 20%, head and neck cancer (15%-25%), colorectal and esophagus cancer (15%-45%), and liver cancer (40%). Awareness was higher among young people and specialized health professions and lower among women (the latter specifically for breast cancer) [83].

### Practical Implications

This study identified a set of variables associated with cancer information seeking. We address 2 implications. First, social media users had higher odds of seeking cancer information. Previous studies found that web-based infotainment videos are an effective approach in increasing public understanding about science and health care among web-based health information seekers and are a useful and effective approach in relaying complex health information, motivating interested viewers to seek additional health information, and driving public audiences to credible and reliable sources of information [86]. Furthermore, social media plays a significant role in how people seek and share information about cancer, both for themselves and for others. While social media can be a valuable tool for connecting with support networks and accessing information, it also presents challenges related to misinformation and the potential for information overload. Despite many perceived benefits of social media use among oncology stakeholders, misinformation poses a critical threat to the value of social media for seeking and sharing cancer-related information [87]. It has been suggested that it is necessary for all key stakeholders—including patients and the public, health care providers, researchers, technology companies, and governmental organizations to proactively address the problem of online health misinformation [87].

Furthermore, awareness and beliefs about alcohol and red meat were significantly associated with cancer information seeking. It has been suggested that knowledge about diet-related cancer risks is essential for behavior change; therefore, increasing public knowledge and risk beliefs about the link between alcohol and cancer, particularly among those who consume alcohol, may contribute to declines in the burden of alcohol-related disease in the United States [84]. Further research is warranted to understand these factors better and to develop effective strategies to improve dietary behaviors among cancer survivors [85].

### Strengths and Limitations

This study has several notable strengths. First, this study used the most recent HINTS 6 data to examine the prevalence of cancer information seeking. The HINTS data provides unparalleled insights into health information seeking behaviors, social media use, beliefs about alcohol and cancer, etc. Second, we performed feature selection using 2 widely used methods, LASSO and Boruta, to identify common variables across both methods. Third, we inferred PC scores (factor scores) and then used weighted LR analyses to estimate the associations of potential factors and PC scores with colorectal cancer screening. Fourth, we compared 5 ML algorithms and found that the RF model demonstrated outstanding classification performance in predicting cancer information seeking. Moreover, we used stepwise LR analysis to confirm the results from ML techniques.

Despite these strengths, our analysis has some limitations. First, because the HINTS data are cross-sectional, we could only identify correlations rather than causal relationships. Future research could address this limitation by applying a rigorous quasi-experimental method to longitudinal datasets. Second, since information from participants was self-reported, our study may be subject to recall bias as well as social desirability bias. Third, a notable limitation is the relatively low response rate of the HINTS 6. Low response rates raise concerns about nonresponse bias, particularly if nonrespondents differ meaningfully from respondents on key variables such as alcohol use, health information–seeking, or cancer awareness. Although HINTS incorporates sampling weights, replicate weights, and nonresponse adjustments to enhance population representativeness, these statistical corrections cannot fully eliminate bias arising from selective participation. Therefore, caution is warranted when interpreting the generalizability of our findings to all US adults. Fourth, the data were collected in 2022, and the COVID-19 pandemic may have influenced both data collection and results. In addition, this study used only the binary outcome (seeker vs nonseeker). For the seekers, there are still 4 questions in the data such as how much do you agree or disagree: it took a lot of effort to get the information you needed, you felt frustrated during your search for the information, you were concerned about the quality of the information, and the information you found was hard to understand. We did not include these subquestions. Furthermore, because the outcome captured lifetime (“ever”) cancer information–seeking, whereas several predictors reflected respondents’ current status in 2022, temporal misalignment may exist. Information seeking may have occurred prior to the measurement of current characteristics; therefore, associations should be interpreted as correlational rather than causal.

### Conclusion

This study provided the updated prevalence of cancer information seeking among US adults. Furthermore, we performed feature selection and compared 5 ML algorithms for classifying cancer information seeking, and identified that RF was the best performer with the highest AUC. Moreover, PCA proved useful for data mining to reduce the indicators in complex survey data and aid in feature selection. In addition, based on the stepwise regression model, 19 out of 27 selected variables were significantly associated with cancer information seeking. We identified a set of predictive variables for cancer information seeking, such as having cancer, having a family history of cancer, worrying about getting cancer, knowledge of genetic tests and HPV, being offered access to health records, higher income, often watching videos on social media, believing that alcohol consumption increases cancer risk, and frequency of visiting providers. Our findings may benefit researchers, policymakers, and health care providers by increasing public awareness and supporting targeted education on cancer information seeking.

## Data Availability

The data that support the findings of this study are openly available at the National Cancer Institute [88].

## Appendix

Multimedia Appendix 1 Feature selection based on LASSO and Boruta. LASSO: least absolute shrinkage and selection operator.

Multimedia Appendix 2 Eigenvalues based on PCA. PCA: principal component analysis.

Multimedia Appendix 3 Rotated component/loading of variables for 20 factors.

Multimedia Appendix 4 Machine learning and comparison of performance.

Multimedia Appendix 5 Additional figures.

## Abbreviations

ANN: artificial neural network
AUC: area under the receiver operating characteristic curve
DT: decision tree
HINTS 6: 2022 Health Information National Trends Survey
HINTS: Health Information National Trends Survey
HPV: human papillomavirus
KNN: k-nearest neighbor
LASSO: least absolute shrinkage and selection operator
LR: logistic regression
ML: machine learning
NCI: National Cancer Institute
OR: odds ratio
PC: principal component
PCA: principal component analysis
RBF: radial basis function
RF: random forest
SVM: support vector machine
XGBoost: extreme gradient boosting
