# Supplementary material for: Comparison of Feature Selection Methods in Machine Learning Models of Cancer Information Seeking Among United States Adults: Cross-Sectional Study
Source: JMIR Med Inform. 2026 Apr 20;14:e75862. doi: 10.2196/75862 (PMC13139833; doi:10.2196/75862)
Supplement: Multimedia Appendix 4 [file medinform_v14i1e75862_app4.docx]

**Table S4.** Machine learning and comparison of performance.

| Model | Variation | Features | Accuracy | Sensitivity (Recall) | Specificity | Precision | F1-Score | AUC |
| --- | --- | --- | --- | --- | --- | --- | --- | --- |
| SVM | Linear kernel | All features | 0.717 | 0.722 | 0.711 | 0.728 | 0.725 | 0.773 |
|  |  | LASSO | 0.709 | 0.717 | 0.701 | 0.720 | 0.719 | 0.780 |
|  |  | Boruta | 0.713 | 0.713 | 0.712 | 0.727 | 0.720 | 0.773 |
|  |  | LASSO and Boruta | 0.711 | 0.715 | 0.706 | 0.724 | 0.720 | 0.777 |
|  |  | PCA score | 0.702 | 0.713 | 0.692 | 0.713 | 0.713 | 0.766 |
|  |  | PCA based - highest loading variable | 0.661 | 0.677 | 0.644 | 0.671 | 0.674 | 0.712 |
|  | RBF kernel | All features | 0.631 | 0.890 | 0.353 | 0.597 | 0.715 | 0.719 |
|  |  | LASSO | 0.701 | 0.831 | 0.564 | 0.756 | 0.792 | 0.759 |
|  |  | Boruta | 0.705 | 0.834 | 0.565 | 0.673 | 0.745 | 0.754 |
|  |  | LASSO and Boruta | 0.708 | 0.778 | 0.633 | 0.695 | 0.734 | 0.771 |
|  |  | PCA score | 0.690 | 0.778 | 0.595 | 0.674 | 0.722 | 0.749 |
|  |  | PCA based - highest loading variable | 0.652 | 0.725 | 0.575 | 0.647 | 0.684 | 0.707 |
| LR | Logistic regression | All features | 0.717 | 0.722 | 0.711 | 0.728 | 0.725 | 0.777 |
|  |  | LASSO | 0.714 | 0.720 | 0.707 | 0.726 | 0.723 | 0.780 |
|  |  | Boruta | 0.714 | 0.722 | 0.704 | 0.724 | 0.723 | 0.775 |
|  |  | LASSO and Boruta | 0.708 | 0.720 | 0.702 | 0.721 | 0.721 | 0.777 |
|  |  | PCA score | 0.705 | 0.719 | 0.691 | 0.714 | 0.719 | 0.767 |
|  |  | PCA based - highest loading variable | 0.664 | 0.679 | 0.648 | 0.674 | 0.676 | 0.712 |
| RF | Random forest | All features | 0.709 | 0.741 | 0.675 | 0.710 | 0.725 | 0.780 |
|  |  | LASSO | 0.714 | 0.740 | 0.686 | 0.716 | 0.728 | 0.781 |
|  |  | Boruta | 0.714 | 0.747 | 0.678 | 0.714 | 0.730 | 0.777 |
|  |  | LASSO and Boruta | 0.711 | 0.740 | 0.679 | 0.712 | 0.726 | 0.779 |
|  |  | PCA score | 0.694 | 0.791 | 0.590 | 0.675 | 0.728 | 0.752 |
|  |  | PCA based - highest loading variable | 0.678 | 0.698 | 0.657 | 0.686 | 0.692 | 0.729 |
| KNN | k-nearest neighbors | All features | 0.665 | 0.531 | 0.801 | 0.744 | 0.619 | 0.737 |
|  |  | LASSO | 0.687 | 0.585 | 0.797 | 0.750 | 0.657 | 0.742 |
|  |  | Boruta | 0.669 | 0.615 | 0.727 | 0.707 | 0.658 | 0.751 |
|  |  | LASSO and Boruta | 0.696 | 0.634 | 0.762 | 0.741 | 0.683 | 0.758 |
|  |  | PCA score | 0.653 | 0.561 | 0.751 | 0.707 | 0.626 | 0.722 |
|  |  | PCA based - highest loading variable | 0.640 | 0.617 | 0.664 | 0.668 | 0.641 | 0.687 |
| XGBoost | Extreme gradient boosting | All features | 0.708 | 0.731 | 0.684 | 0.713 | 0.722 | 0.768 |
|  |  | LASSO | 0.712 | 0.731 | 0.693 | 0.719 | 0.725 | 0.771 |
|  |  | Boruta | 0.714 | 0.736 | 0.691 | 0.719 | 0.727 | 0.765 |
|  |  | LASSO and Boruta | 0.697 | 0.711 | 0.674 | 0.703 | 0.707 | 0.767 |
|  |  | PCA score | 0.685 | 0.716 | 0.650 | 0.688 | 0.702 | 0.745 |
|  |  | PCA based - highest loading variable | 0.656 | 0.704 | 0.657 | 0.655 | 0.679 | 0.724 |

Abbreviations: SVM: Support vector machine; RBF: Radial basis function; AUC: Area under the ROC (receiver operating characteristics) curve; LASSO: The least absolute shrinkage and selection operator; PCA: Principal component analysis.
