# Supplementary material for: Comparison of Feature Selection Methods in Machine Learning Models of Cancer Information Seeking Among United States Adults: Cross-Sectional Study
Source: JMIR Med Inform. 2026 Apr 20;14:e75862. doi: 10.2196/75862 (PMC13139833; doi:10.2196/75862)
Supplement: Multimedia Appendix 5 [file medinform_v14i1e75862_app5.docx]

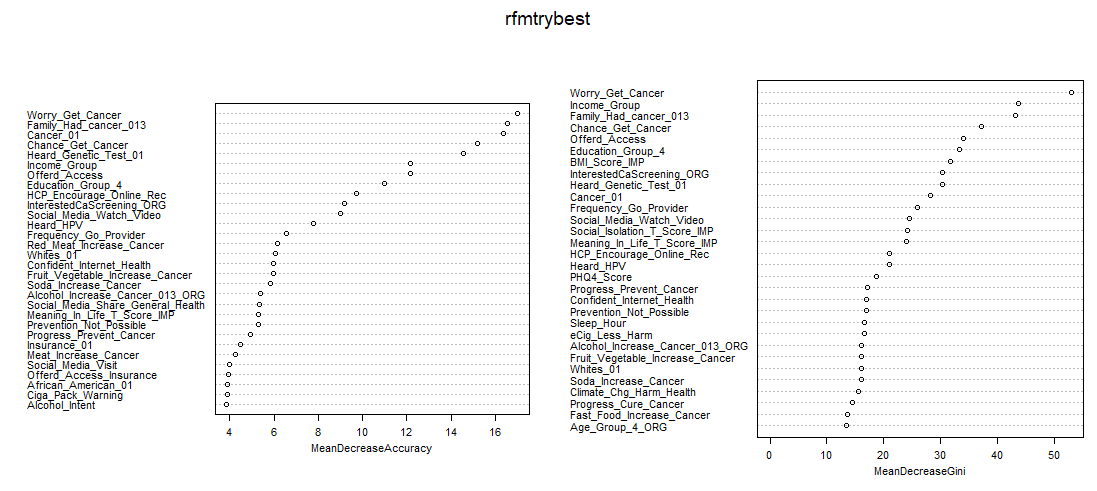


**Figure S1**. Plot of mean decrease accuracy (left panel) and mean decrease gini (right panel) values using random forest algorithm and top 30 of 73 variables


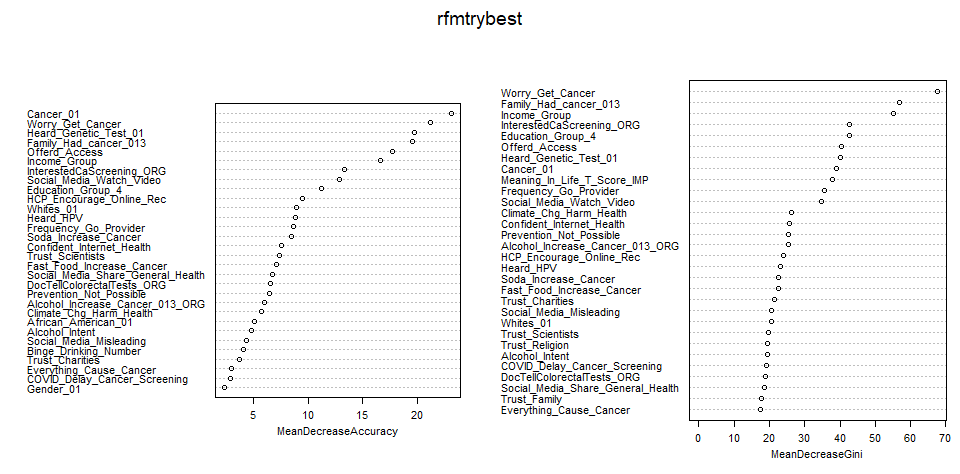


**Figure S2**. Plot of mean decrease accuracy (left panel) and mean decrease gini (right panel) values using random forest algorithm and top 30 of 42 variables selected by LASSO


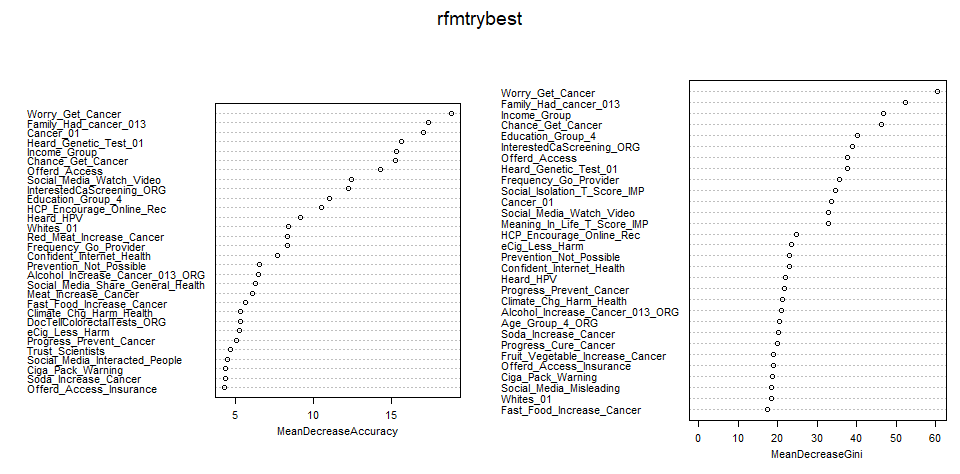


**Figure S3**. Plot of mean decrease accuracy (left panel) and mean decrease gini (right panel) values using random forest algorithm and top 30 of 43 variables selected by Boruta


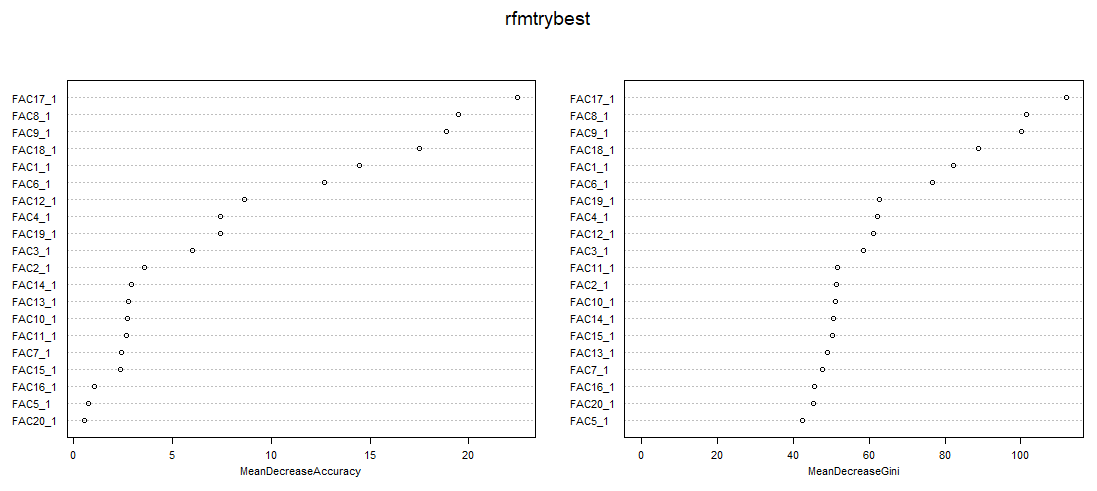


**Figure S4**. Plot of mean decrease accuracy (left panel) and mean decrease gini (right panel) values using random forest algorithm and 20 factors
